# Closing evidence gaps in general practice & family medicine. Selected Abstracts from the 92nd EGPRN Meeting – Virtual Conference, 30 April to 1 May 2021: All abstracts of the conference can be found at the EGPRN website https://www.egprn.org/page/conference-abstracts

**DOI:** 10.1080/13814788.2021.2008352

**Published:** 2021-12-13

## Introduction

Patients rightly expect us to provide them with the best possible medical treatment. Therefore, we have increasingly used and refined the principles of evidence-based medicine over the past two to three decades in our research. In the recent past, the evidence published in scientific journals and elsewhere has started to grow at an unprecedented pace, now nearly doubling every 4 years. On average, a new medical article is published every 15 s. To a growing extent, the evidence informing our clinical decision-making comes from our very own setting. However, there is not only a change in quantity: a growing number of today’s clinical trials also encompass patients’ preferences and include relevant details needed for implementation in clinical decision making.

Increasing the quantity of evidence can in itself create a variety of new problems: health care professionals are challenged to remain up to date with new evidence like never before. Systematic reviews show that adherence to guideline recommendations in daily routine care varies from 20% to over 80%. Thus, despite rapidly growing knowledge, a varying share of patients are still likely to receive suboptimal treatments, inappropriate diagnostics, unsafe medications, and costly but ineffective care. Incorporating new evidence into daily practice already usually takes several years. This ‘evidence to practice gap’ might even get bigger the more evidence there is.

At the same time, we still face an ongoing lack of evidence in other domains. This is partly due to the characteristics of research in primary care, for example, varying organisational structures, practice team compositions, or different contexts caused by the health care system and its legal boundaries. Besides these contextual factors, research in primary care frequently deals with complex interventions and patients with very heterogeneous characteristics, which leaves us with many potential sources of uncertainty when we try to put together guidelines based on evidence in our field of research.

Fortunately, in the wake of digitisation, new and promising ways of combining and integrating evidence, for example, learning health care systems, big data analysis and machine learning, are at our disposal. Nonetheless, closing evidence gaps is an ongoing challenge that is only partially solved by creating new or more evidence or combining more and more information. In fact, we still have to perform constantly assess evidence gaps, both in generating evidence and in translating evidence into practice. Ideally, a prioritisation of research questions follows this assessment and, in addition, existing evidence gaps should be addressed in trials in real-world conditions.

During the 92nd EGPRN meeting, a virtual conference on 30 April and 1 May 2021, gaps in evidence and ways to study evidence gaps in general practice and family medicine were discussed.

