# Supplementary material for: Closing evidence gaps in general practice & family medicine. Selected Abstracts from the 92nd EGPRN Meeting – Virtual Conference, 30 April to 1 May 2021: All abstracts of the conference can be found at the EGPRN website https://www.egprn.org/page/conference-abstracts
Source: Eur J Gen Pract. 2021 Dec 13;27(1):354–65. doi: 10.1080/13814788.2021.2008352 (PMC8754170; doi:10.1080/13814788.2021.2008352)
Supplement: Related texts

# Abstract: What matters most? Development of a research agenda for general practice

**DOI:** 10.1080/13814788.2021.2008352

**Authors:** Jako Burgers

**Affiliations:** Department of General Practice and Care and Public Health Research Institute of Maastricht University, Maastricht, The Netherlands

## Abstract

Research in general practice covers the whole of medicine, including clinical and contextual issues. Defining the focus of research is, therefore, not easy. Knowledge and preferences of researchers and funding agencies may dominate above the real problems faced by patients and society. In addition, topic selection and prioritisation are often not coordinated, leading to duplication and research waste. A research agenda could help focus on the issues that matter most.

In the Netherlands, we developed a national research agenda involving general practitioners (GPs), researchers, patients and other relevant stakeholders in healthcare. We reviewed knowledge gaps from 90 Dutch general practice guidelines and received input from 48 healthcare stakeholders to formulate research questions relevant for general practice. This resulted in a long list of 787 research questions. These were prioritised by practising GPs (*n* = 232) in an online survey and in an invitational conference, including GPs (*n* = 48) and representatives of other stakeholders in healthcare (*n* = 16). The prioritising procedure resulted in 24 top 10 lists of research questions categorised according to ICPC chapters and overarching themes such as person-centred care, eHealth, and organisation of care. An advisory board composed of general practice research departments representatives, the Dutch College of GPs, GP trainees, and patient organisations supported the whole process.

The national research agenda provided an enormous boost in general practice research as well as in collaboration between research departments. A national general practice research programme has been launched to address the questions in the agenda. Collaboration instead of competition between research departments and institutes is encouraged within this programme. They are united in a national consortium supported by the Dutch College of GPs to facilitate collaboration between data registration networks and initiate large-scale multicentered trials.

The Dutch general practice research agenda could be an example for other countries and international collaboration. Opportunities to collaborate within the EGPRN to set up an international research agenda are challenging. This could include cross-border themes such as infectious diseases, migrant care, and planetary health. A shared agenda could also increase the chances of obtaining European funding. It is time now for international collaboration in general practice research contributing to health that matters to all.

# Abstract: Digital technologies: Opportunities and challenges for better, safer and more equitable primary care

**DOI:** 10.1080/13814788.2021.2008352

**Authors:** Ana Luisa Neves

**Affiliations:** Center for Health Technology and Services Research, University of Porto, Porto, Potugal

## Abstract

Digital technologies may improve the quality of primary care by improving patient empowerment, access to personal health information and increased involvement in the self-management of their health and disease. However, with the widespread use of these digital solutions, there is a growing need to evaluate their impact to better understand their risks and benefits and inform health policies that are both patient-centred and evidence-based. It is therefore critical to evaluate their impact and map it against the six domains of healthcare quality: patient-centredness, effectiveness, efficiency, safety, timeliness and equity.

Using digital platforms such as patient portals and mobile apps as tools to provide patients with access to their health information can positively impact health outcomes (i.e. glycemia levels) and improve patient safety outcomes, such as general adherence and medication safety. However, more studies are necessary to enhance meta-analytical power and assess the impact in other care domains.

For decades, digital technologies have promised to help address many challenges in primary care. Around the world, countless initiatives have made considerable efforts to implement remote care approaches into existing healthcare systems. However, despite the promise, a wide range of barriers have limited widespread adoption, including cultural, regulatory and policy, social security, industrial and technical, knowledge, financial, and market-related barriers.

With the onset of COVID-19, we experienced another radical transformation in primary care - the shift to remote care. Over the course of a few weeks, primary care physicians and patients worldwide swiftly transitioned from face-to-face consultations to remote solutions. Remote care has reduced risks of viral transmission by deflecting patient flow from healthcare physical facilities, whilst simultaneously allowing physicians to continue providing care to vulnerable patients who require shielding. It has supported the triage of patients with COVID-19 related symptoms (potentially optimising the use of limited healthcare resources), supported monitoring of COVID-19 patients, and strengthened epidemiological surveillance efforts. In addition to COVID-19-related care, digital tools have also contributed extensively to the provision of care for both chronic diseases and, in some circumstances, acute care. However, some concerns have been raised, particularly concerning potential safety issues regarding the monitoring COVID-19 patients, and worsening health inequalities for patients who are not technologically literate or have hearing impairment.

As primary care embraces technology (i.e. with virtual care and telemedicine), there are important concerns about the impact of digital approaches widening the digital divide, that is, entrenching healthcare disparities for low-income, racial-ethnic minority and linguistically diverse populations. Therefore, it is critical that future research widely evaluates patient interest in, access to and skills for using digital care tools, leading to the development of tailored interventions to match patient preferences and needs. In this context, mixed-methods approaches and implementation science studies can be an important tool to understand use, usability, and uptake, as well as the impact on health outcomes.

# Abstract: Adoption and evaluation of a completely digital general practice clerkship – Cross-sectional survey and cohort comparison among German medical students

**DOI:** 10.1080/13814788.2021.2008352

**Authors:** Marina Fehl, Vera Gehres, Anne-Kathrin Geier, Thomas Mundt, Kay Klinge, Markus Bleckwenn, Tobias Deutsch

**Affiliations:** Department of Family Medicine, Selbstständige Abteilung für Allgemeinmedizin, Leipzig, Germany

## Abstract

**Background:**

During the COVID-19 pandemic, the University of Leipzig completely switched to online teaching. Thus, we developed a practice-oriented digital equivalent of a mandatory 2-week general practice (GP) clerkship. The digital clerkship mainly contained clinical cases, visual diagnoses, informational and examination videos, as well as regular possibilities for exchange (e.g. video chat) with associated GP teachers in their practices, faculty members and fellow students.

**Research question:**

How did the participants accept, use and evaluate the new format and its single components regarding working enjoyment, learning gain, practical relevance and insights into general practice? How do evaluations differ from those of two previous semesters?

**Methods:**

Cross-sectional survey among 4th year (of six) medical students at Leipzig medical faculty completing their digital mandatory GP clerkship between April and June 2020. Additional cohort comparison with evaluations of two previous semesters.

**Results:**

Out of 192 students who completed the digital clerkship, 99 participated in the study (51.6%). For the cohort comparison, evaluations from 277 students of the two previous semesters (conventional clerkship) were available. Most participants reported to have enjoyed the online-based clerkship (88%), to have learned a lot (90%), to have gained insights into general practice (77%) and perceived high practical relevance (91%). Two-thirds stated that the new format should complement future clerkships. Clinical cases, visual diagnoses, examination videos and communication with GP teachers were rated best regarding working enjoyment, learning gain, practical relevance and insights into a GP’s work. Cohort comparison revealed partially better evaluations regarding knowledge transfer for the digital clerkship while imparting of skills and attitudes was evaluated worse.

**Conclusion:**

Our digital teaching formats were well accepted by the students. In particular, learning from selected clinical cases, visual diagnoses, video tutorials, and explicit slots for exchange with GP teachers and faculty members have a high potential to complement future conventional clerkships usefully.

# Abstract: Is continuity of care associated with use of health care services?

**DOI:** 10.1080/13814788.2021.2008352

**Authors:** Emmi Lautamatti, Markku Sumanen, Risto Raivio, Kari Mattila

**Affiliations:** Department of General Medicine, Tampere University, Tampere, Finland

## Abstract

**Background:**

Continuity of primary health care is a defining characteristic of high-quality health care. There is evidence of continuity of care having associations with the use of health care services. In Finland, continuity of care in primary care has decreased and at the same time the use of hospital services, examinations and consultations have increased.

**Research question:**

Is continuity of care associated with use of health care services? Does continuity of care increase the quality of care by decreasing unnecessary examinations and consultations? Is there a patient group that benefits from continuity of care more than others?

**Methods:**

The data are part of the Health and Social Support study (HeSSup) based on a random Finnish population sample. There were 11,924 respondents in 2003 and 15,993 in 2012. Participants were divided into three groups according to the single repeated question of continuity of care. We asked about the use of laboratory tests, consultations or other examinations in the questionnaires. Logistic regression analysis was used to study the association of age, gender, education, self-assessed health, ability to function, chronic diseases and continuity of care with more frequent use of health care services.

**Results:**

Participants in the group who had continuity of care in 2003 and 2012 had more illnesses than participants in other groups. Also, in this group, the use of other examinations, consultations and hospital services was more frequent than in other groups. Factors associated with the findings are still under study. The results will be announced in the congress.

**Conclusion:**

Patients with chronic diseases need and usually get more health care services than the rest of the population. Patients pursue authorities to implement continuity of care, which also guarantees good quality of care.

# Abstract: General practice attendances among patients attending a post-COVID-19 hospital clinic: A pilot study

**DOI:** 10.1080/13814788.2021.2008352

**Authors:** John Broughan, Geoff Mccombe, Gordana Avramovic, Des Crowley, Cheyenne Downey, Joanne Downey, Ronan Fawsitt, Tina Mchugh, Eileen O’connor, Carla Perrotta, Aoife Cotter, John S Lambert, Walter Cullen

**Affiliations:** School of Medicine, Primary Care Research Group, University College Dublin, Dublin, Ireland

## Abstract

**Background:**

About 10–35% of people with COVID-19 merit medical care within 3 weeks of infection. However, the prevalence of ongoing care needs among individuals experiencing severe COVID-19 illness is unclear.

**Research question:**

What is the prevalence of ongoing care needs among severe COVID-19 patients?

**Methods:**

This pilot study applied a cross-sectional design whereby data was collected from adult patients attending a post-COVID-19 follow-up clinic at the Mater Misericordiae University Hospital, Dublin, Ireland, 3–6 months after their initial presentation at the clinic. Participants completed questionnaires documenting their demographics, medical histories, hospital admissions/re-admissions where applicable, and where relevant, primary care service use following hospital discharge. Analyses were conducted using descriptive/inferential statistics.

**Results:**

Participants’ (*n* = 153) median age =43.5 (IQR =30.9–52.1). There were 105 females (68.6%) and 48 males (31.4%). Various medical histories were reported among participants. 67 (43.2%) reported being admitted to the hospital for COVID-19. Older individuals, males, ICU admissions, and re-admissions were common among hospital attendees. Of the hospital attendees, 16 (24%, 95% CI =13.7–34.2%) and 26 (39%, 95% CI =27.3–50.7%) attended general practices within seven and 30 days of hospital discharge. Older adults (median age =49.8 years), people with pre-existing medical conditions, and individuals admitted to ICU/readmitted to hospital were common among general practice attendees.

**Conclusion:**

Persistent health issues appear to be common among patients who experienced severe COVID-19 illness. Older adults, people with pre-existing health problems, and individuals who received ICU and/or re-admission care may have greater long-term care needs requiring attention.

# Abstract: Demographic evaluation of hospital staff before Covid vaccination

**DOI:** 10.1080/13814788.2021.2008352

**Authors:** Gökçe İşcan, Funda Yildirim Baş

**Affiliations:** Department of Family Medicine, Suleyman Demirel Üniversitesi, Isparta, Turkey

## Abstract

**Background:**

Despite the strong value of vaccination in reducing the global burden of infectious diseases, the anti-vaccination movement is growing. Our hospital staff in Turkey were one of the first groups to be vaccinated, and we wanted to explore if there were any biases in attitudes toward vaccination.

**Research question:**

Is there a relationship between the hospital staff’s COVID-19 status and their desire to be vaccinated? What are the reasons provided by staff who do not want to be vaccinated?

**Methods:**

We designed a web-based survey focussing on demographic information, working status, desire to be vaccinated, COVID-19 status and chronic disease history. The survey was sent to 2000 staff and students (4th to 6th-year medical students), 949 people returned the survey. Data were analysed using SPSS-22. The chi-square test was used for comparisons.

**Results:**

47.45% (949) of 2000 staff answered the survey of those aged 18–61 years (32.4 ± 9.79). 501 of them were women (52.8%); 448 of them were men (47.2%); 72 had a history of COVID-19 infection (7.6%); 27 (2.8%) had suspicious COVID-19 contact in the past 14 days. A total of 925 participants (97.5%) wanted to be vaccinated and 24 (2.5%) did not. 357 participants (37.6%) had a chronic disease, most common were hypertension (*n* = 56, 27.1%), lung disease (*n* = 42, 11.8%), hypothyroidism (*n* = 40, 11.2%) and diabetes mellitus (*n* = 32, 9%). Women got COVID-19 more than men and it was statistically significant (*p* = 0.001). There was no statistically significant difference between age, gender, department or having a chronic disease and wanting to be vaccinated (*p* < 0.05).

**Conclusion:**

Only 24 of 949 people did not want to be vaccinated. This is a deficient proportion because all of them worked in the hospital as students, doctors or employees. Most of them know/see the importance of COVID-19 infection. The 24 people who did not want to be vaccinated, neither had previous COVID-19 infection nor gave any other stated reasons. It is thought-provoking.

# Abstract: Post-lockdown impact of the COVID-19 pandemic on patients affected by chronic diseases in Europe

**DOI:** 10.1080/13814788.2021.2008352

**Authors:** Alexis Astruc, Bruno Halioua, Jean Zetlaoui, Meryem Harrizi, Alexis Bombezin–Domino, Lise Radoszycki

**Affiliations:** Department of General Practice, University Sorbonne Paris Nord, Bobigny, France

## Abstract

**Background:**

Patients with Chronic Medical Conditions (CMC) require regular access to healthcare professionals and take long-term treatments. The COVID-19 pandemic may affect their clinical outcomes.

**Research question:**

What is the impact of the COVID-19 epidemic on patients with CMC in Europe?

**Methods:**

This observational cross-sectional study was conducted using an online questionnaire available on a social network of patients (Carenity) from 3 June 2020 to 7 October 2020. It included adult patients from France, Germany, Italy, Spain and the United Kingdom, with any CMC.

**Results:**

A total of 2861 patients were included, 75% woman (*n* = 2136), with a mean age of 54 years old, and most frequently affected by asthma (22%, *n* = 619), type 2 diabetes (16%, *n* = 467), or chronic obstructive pulmonary disease (12%, *n* = 332), 89% (*n* = 2538) of them were taking a long-term treatment, 17% (*n* = 433) modified their treatment intake, 30% (*n* = 132) without notifying their doctor. Consultations were strongly impacted: from the start of the epidemic, 30% (*n* = 872) of patients had difficulty in finding an available doctor, and 28% (*n* = 794) consulted their doctor less frequently since end of lockdown (45%, *n* = 1287, during lockdown). 75% (*n* = 2135) of patients had long-planned medical consultations or procedures cancelled because of the epidemic. Of those, 63% (*n* = 1343) could not reschedule all of them. 39% (*n* = 1109) feel their health status has deteriorated because their disease management was modified during lockdown. 34% (*n* = 973) of patients used tele-consultation: 80% (*n* = 782) of them deemed it satisfactory and 59% (*n* = 570) were willing to use it for their future CMC follow-up. Patients also reported lacking information, mainly regarding treatment-related risks and precautions when returning to work. 46% (*n* = 1316) were not satisfied with the information they received.

**Conclusion:**

COVID-related changes in CMC patients’ behaviour and medical care may have health consequences. They should be closely monitored to make sure CMC patients are not the collateral victims of the epidemic.

# Abstract: Usability of a communication tool used by general practitioners and public health workers during the COVID-19 pandemic: A proof-of-concept-study

**DOI:** 10.1080/13814788.2021.2008352

**Authors:** Elliott, Anika Thielmann, Beate Müller, Corina Guethlin, Ferdinand Gerlach, Angelina Müller, Pascal Nohl-Deryk, Sylvia Schulz-Rothe, Phillip Elliott, Anika Thielmann, Beate Müller, Corina Guethlin, Ferdinand Gerlach

**Affiliations:** Institute of General Practice, Goethe-University Frankfurt, Frankfurt, Germany

## Abstract

**Background:**

With rising numbers of Covid-19 patients and patients requiring testing and quarantine, administrative burden increased and new challenges for existing communication pathways emerged. While at the beginning of the pandemic, communication between local health authorities and general practitioners in Germany heavily relied on telephone and fax machines, resources for the exchange of real-time information were limited. To explore whether an instant messaging app could affect communication structures between local healthcare players in the pandemic, we conceptualised a proof-of-concept study testing deployment of a communication tool in two regions in Germany. The study is funded by the German Federal Ministry for Research and Education and is part of the COVID-19-national research network.

**Research question:**

To identify the experiences of general practitioners and local health authorities with an app for safe instant communication.

**Methods:**

We conducted a proof-of-concept study comprising implementation and evaluation of an instant messenger app used by general practitioners, local health authorities and COVID-specialists in the Rhein-Main-area in Germany. Participants started to use the app in November 2020. Online questionnaires are used to assess usability. We used a pretested German translation of the mobile health app usability questionnaire (MAUQ).

**Results:**

Participants will use the app in the following weeks and subsequently rate it. Results will be available at the congress.

**Conclusion:**

The results can provide further insights into the suitable deployment of communication apps in healthcare settings.

# Abstract: Frequency of occurrence and predictive validity of olfactory and taste dysfunction in patients with SARS-CoV-2 infection

**DOI:** 10.1080/13814788.2021.2008352

**Authors:** Esperanza Romero Rodríguez, Luis Ángel Pérula De Torres, Jesús González-Lama, Celia Jiménez García, Faustino Rider Garrido, Yolanda Ortega López, Diego Pajares Conde, Miguel Ramírez Baena, Ignacio Párraga Martínez, Grupo Colaborativo Epicovid, Rosa Sánchez Montero

**Affiliations:** IMIBIC, Córdoba, Spain

## Abstract

**Background:**

Olfactory and taste dysfunction (OD and TD) have been considered symptoms of SARS-CoV-2 infection. Although these clinical features may occur prior to the general symptoms of such infection, their presence in specific populations, especially those with mild symptoms, has not been clarified. This study aimed to estimate the frequency of OD and TD, and its predictive validity in patients with SARS-CoV-2 infection detected in primary care.

**Research question:**

What is the frequency of OD and TD, and its predictive validity in patients with SARS-CoV-2 infection detected in primary care?

**Methods:**

A cross-sectional study was carried out in the Spanish National Health System through an epidemiological survey administered to patients who required an RT-PCR test (real-time polymerase chain reaction in a nasal/pharyngeal swab) to detect SARS-CoV-2. Odds Ratio(OR)(s) were estimated to measure the magnitude of the association between OD or TD, and the presence of SARS-CoV-2 infection. The study sample also calculated the sensitivity, specificity, and positive and negative predictive values (PPV, NPV) of these symptoms.

**Results:**

Of 1038 patients screened, 209 (20.1%) had SARS-CoV-2 infection. OD and DG were detected in 64.4% (95% CI: 56.0–72.1) and 56.2% (95% CI: 47.9–64.2) of subjects with infection, respectively. The OR for OD and TD was 12.2 (95% CI: 8.26–18.06) and 7.95 (95% CI: 5.48–11.53), respectively. OD showed a sensitivity of 45.0% (95% CI: 37.6–51.5), a specificity of 93.7% (95% CI: 91.8–95.0), a PPV of 64.4% (95% CI: 56.0–72.1), and a NPV of 87.1% (95% CI: 84.7–89.2) while the TD presented a sensitivity of 41.1% (95% CI: 34.4–46.1), a specificity of 91.9% (95% CI: 89.8–93.7), a PPV of 56.2% (95% CI: 48.0–64.2) and a NPV of 86.1% (95% CI: 83.6–88.3).

**Conclusion:**

More than half of the subjects with SARS-CoV-2 infection have olfactory or taste dysfunction. These clinical features could be considered of diagnostic utility due to their ability to predict infection in more than half of the cases.

# Abstract: Cardiometabolic health reviews in patients on antipsychotics: The impact of Covid-19

**DOI:** 10.1080/13814788.2021.2008352

**Authors:** Simon Erridge, Christopher Watts

**Affiliations:** York Medical Practice, London, UK

## Abstract

**Background:**

Antipsychotics are associated with metabolic disturbances adversely affecting cardiometabolic health. Routine cardiometabolic monitoring is subsequently an important component of care in patients on antipsychotics. During the Covid-19 pandemic, there has been a transition towards remote consulting. Understanding the effect of the pandemic on routine primary care activity may help avoid potential adverse health outcomes.

**Research question:**

This study aimed to identify the local impact of the Covid-19 pandemic on cardiometabolic monitoring in patients on antipsychotics.

**Methods:**

A cross-sectional analysis was performed of patients on antipsychotic medications at a suburban South-West London practice. Patients were identified through a search via EMIS Web. Inclusion criteria included those registered permanently and commenced on medication prior to 17 December 2018. Primary outcomes included the difference in the yearly incidence of recorded weight, waist circumference, pulse, blood pressure, fasting blood glucose, HbA1c and lipids. Statistical significance was defined by *p* < 0.050.

**Results:**

Fifty-three patients met the inclusion criteria. The mean patient age was 52.2 (±17.4). A total of 64 antipsychotics were on repeat prescriptions, with quetiapine (*N* = 18; 28.1%) and olanzapine (*n* = 18; 28.1%) being the most commonly prescribed. Comparing 2019 against 2020, there was no statistically significant difference in incidence of recorded weight (2019: 49.2%; 2020: 49.2%; *p* = 0.698), waist circumference (2019: 10.2%; 2020: 6.8%; *p* = 0.486), pulse (2019: 20.3%; 2020: 11.9%; *p* = 0.672), blood pressure (2019: 59.3%; 2020: 44.1%; *p* = 0.821), fasting blood glucose (2019: 5.1%; 2020: 0.0%; *p* = 1.000), HbA1c (2019: 72.9%; 2020: 49.2%; *p* = 0.613), lipids (2019: 55.9%; 2020: 39.0%; *p* = 0.251).

**Conclusion:**

A reduction in cardiometabolic monitoring was observed in this studied population locally in 2020. This was not statistically significant but may be clinically significant for long-term health outcomes on an individual patient basis. It is important that any potential adverse effects on different populations of increased remote consulting during and beyond the Covid-19 pandemic are identified. Health systems may be reconfigured to ensure robust follow-up and reduce excess mortality.

# Abstract: Are we sitting too comfortably? What evidence do we have regarding sedentary behaviour among GPs?

**DOI:** 10.1080/13814788.2021.2008352

**Authors:** Richard Mayne, Nigel Hart, Neil Heron

**Affiliations:** Queen’s University Belfast, Belfast, UK

## Abstract

**Background:**

Excessive sedentary behaviour is associated with several adverse health outcomes and increased all-cause mortality. GPs who are more physically active are more likely to recommend physical activity to their patients.

**Research question:**

What is the current evidence regarding levels of sedentary behaviour among GPs?

**Methods:**

A systematic review was conducted to establish the evidence regarding current levels of sedentary behaviour among GPs. Subsequently, a multi-item questionnaire survey (modified version of the International Sedentary Assessment Tool (ISAT)) was disseminated to GPs in Northern Ireland. A purposive, maximally varied sample of 20 survey participants was then recruited to wear thigh-worn accelerometers and complete a sleep/work log to obtain objective data regarding their sedentary behaviour. This allowed the comparison of subjective, self-reported data with objective, accelerometer data.

**Results:**

Systematic Review: Search criteria returned 1707 studies. 34 full texts were reviewed and two studies were included in the final review. Both were cross-sectional surveys of satisfactory methodological quality and a high risk of bias. Sedentary Behaviour Study: Out of 1999 GPs in Northern Ireland, the questionnaire received 352 valid responses (response rate of 18%). Overall mean workday sedentary time for GPs was 10 h 20 min. Overall mean non-workday sedentary time was 4 h 47 min. Only 6% of GPs had access to an active workstation, such as a standing desk, however, 61% of those who didn’t have an active workstation would consider using one. 81% of GPs reported spending more time sitting at work now than before the COVID-19 pandemic. 87% of GPs would prefer less time sitting at work.

**Conclusion:**

Sedentary behaviour among GPs has increased since the onset of the COVID-19 pandemic, with the vast majority of GPs exceeding the recommended daily levels of sedentary behaviour. Further research is required to identify ways of reducing sedentary behaviour and increasing physical activity among GPs.

# Abstract: Adaptation of the vaccine hesitancy scale to Turkish: Validity and reliability study

**DOI:** 10.1080/13814788.2021.2008352

**Authors:** Kübra Temel Aslan, Cigdem Apaydin Kaya, Pınar Ay

**Affiliations:** Department of Family Medicine, Marmara University School of Medicine, İstanbul, Turkey

## Abstract

**Background:**

A standard, valid measurement tool that assesses vaccine hesitancy will help develop research and immunisation policies, identify individuals with vaccine hesitancy, and overcome hesitations. This study aimed to adapt a Turkish translation of the Vaccine Hesitancy Scale formed by the WHO SAGE Vaccine Hesitancy Working Group.

**Research question:**

Is the Vaccine Hesitation Scale a valid and reliable scale in Turkish society?

**Methods:**

This is a reliability and validity study. The study was carried out with parents of 0- to 18-month-old children who applied to a Training Family Health Center in Istanbul. After the Likert-type Vaccine Hesitancy Scale (answers from 1 to 5, high scores indicate low vaccine hesitation) was translated into Turkish, a test-retest method and Cronbach alpha coefficient were used for reliability research then exploratory factor analysis was used to construct validity.

**Results:**

No statistical difference was found between test-retest scores of the scale (43.55 ± 4.5; 43.70 ± 4.4; *r* = 0.97; *p* < 0.001). Cronbach alpha coefficient was 0.73. Two factors emerged as a result of the exploratory factor analysis: ‘lack of confidence’ and ‘risk perception.’ Mothers were more hesitant than fathers (*p* = 0.005); non-working mothers were more hesitant than working mothers (*p* = 0.026). Those with high income were less hesitant than those with low income (*p* = 0.002). Fathers between the ages of 30–39 years were more hesitant than fathers between 18 and 29 years (*p* = 0.019).

**Conclusion:**

Our results suggest that the Turkish version of the Vaccine Hesitancy Scale is a reliable and valid scale. The VHS can be used for Turkish parents to assess vaccine hesitancy.

# Abstract: Developing and tailoring a complex intervention for the primary prevention of cardiovascular disease prior to its implementation in general practices in Belgium

**DOI:** 10.1080/13814788.2021.2008352

**Authors:** Naomi Aerts, Sibyl Anthierens, Kathleen Van Royen, Lieve Peremans, Peter Van Bogaert, Hilde Bastiaens

**Affiliations:** Department of Primary and Interdisciplinary care, University of Antwerp, Wilrijk, Belgium

## Abstract

**Background:**

Cardiovascular diseases (CVDs) are the world’s leading cause of mortality. CVD and risk factors can be prevented by addressing unhealthy lifestyle behaviours. In Belgium, contextual factors pose a burden on primary health care and despite national guidelines, systematic implementation of prevention is lacking. This work is part of the Horizon 2020 project ‘SPICES’, which aims to implement risk profiling and communication, and behavior change counseling.

**Research question:**

We aimed to design and tailor an evidence-based complex intervention for the primary prevention of CVD, prior to its implementation in general practices in vulnerable city districts in Belgium.

**Methods:**

Development of the intervention was based on a systematic review of international guidelines and a contextual analysis with stakeholder interviews. Acceptability, adoption and appropriateness were assessed through interviews and implementation strategies were tailored to the resources, needs and preferences of the implementers at the general practice level. Our participatory action research design allows ongoing process evaluation throughout the implementation to adapt the intervention or strategies where needed.

**Results:**

The intervention is designed to support awareness of CVD risk and modification and maintenance of healthy lifestyle behaviours to decrease individual CVD risk. We developed a guidebook to communicate the individual CVD risk as a result. The behaviour change counselling component consists of ten lifestyle coaching sessions with set intervals, delivered by a practice nurse over 1 year, interacting with the available community resources. Given the implementers’ concerns around intervention complexity and competence gap, one of the most important implementation strategies applied is the training of all providers of one or more components of the intervention, prior to the implementation.

**Conclusion:**

This study provides a practical example of translating evidence into practice, outlining the development and tailoring of an evidence-based intervention designed in co-creation with multi-level stakeholders to reduce the risk of CVD.

# Abstract: The evaluation of diffuse or focal thyroid pathology and early diagnosis of thyroid malignancy performed by family physicians using artificial intelligence through a European cross-border multicenter ultrasound screening project

**DOI:** 10.1080/13814788.2021.2008352

**Authors:** Mihai Iacob

**Affiliations:** Advitam Medicis Medical Center, Family Medicine and Pediatrics, the Ultrasound Working Group – Euvekus, Timisoara, Romania

## Abstract

**Background:**

This project has three stages. The first step was the development of a smart computerised diagnostic algorithm used to stratify the risk in thyroid pathology, ultrasound-based. It set the optimum time to achieve a thyroid biopsy (FNAB). We have used the latest international classifications (two international scores: EU-TIRADS/ACR_TIRADS), as well as a scoring made by us, correlated with the pathological results. The second stage included a Targeted Thyroid Screening in a high-risk population. Finally, we are launching a cross-border, interdisciplinary, multicenter US Screening.

**Research question:**

How can we diagnose thyroid malignancies early in a high-risk population in primary healthcare by using new medical technology and artificial intelligence?

**Methods:**

We report a targeted thyroid screening performed on 4386 – apparently healthy – adults with oncological risk factors+, aged over 20 years, followed for 5 years. We used the TIRADS classification by Russ modified and Strain Elastography, with both the elastographic scores by Rago and semiquantitative Strain Ratio (SR), for standardisation and to establish if fine needle aspiration biopsy (FNAB) should be performed. The positive patients with focal thyroid lesions found at this screening by family doctors were validated by endocrinologists through ultrasonography, FNAC, and histopathological or cytological examination. We designed an Ultrasound Scoring System (USS) for predicting malignancy and diagnostic algorithm software. All patients were stored and counted into a Smart Thyroid Ultrasound Software. Finally, we compared ultrasound scores designed by us with the histological results as the gold standard method.

**Results:**

This study found 861 patients with thyroid diffuse disease and 696 with focal lesions. Prevalence of thyroid pathology was 38.99% (95% CI: 37.54–40.45%) with screening sensitivity: 96.49% specificity: 96.52% and a high accuracy of 96.51%, PPV: 94.66%, NPV: 97.73%, statistically significant, *p* < 0.01. The ROC analysis of our US methods confirmed a higher level of diagnostic accuracy of Strain Elastography, *p* < 0.001, AUC =0.995, 95% CI: 0.97–1. Our cut-off value of SR was 2.5.

**Conclusion:**

Performing Doppler Triplex Ultrasound Screening together with Strain Elastography had the best accuracy for the analysis of the vascular network and the tumour stiffness, for differentiating ’benign versus malignant thyroid tumours and for diagnosis of diffuse thyroid diseases by family physicians with use of artificial intelligence as a support tool for risk stratification.

# Abstract: Privacy by design and data minimisation in RADAR project.

**DOI:** 10.1080/13814788.2021.2008352

**Authors:** Johannes Hauswaldt

**Affiliations:** Institute of General Practice, University Medical Center Göttingen, Göttingen, Germany

## Abstract

**Background:**

Primary care practice (PCP) teams’ inclusion at the micro-level research demands building and maintaining a complex research infrastructure. Privacy protection and data security have to be considered and incorporated from the starting point when using PCPs’ computerised medical records (EMR).

**Research question:**

Which concepts for privacy protection have been realised during the RADAR project, and which are to be kept or extended in future practice-based research networks?

**Methods:**

Concept analysis and artifact evaluation.

**Results:**

The multi-professional RADAR project team generated and followed comprehensive privacy protection and IT-security concept. It named responsible controllers and time limits for data storage. Of four different data access models, restricted access was realised in the RADAR project, but in the future may be replaced by controlled access. Privacy by design was included in the RADAR project’s conceptualisation right from the beginning, and its 7 foundational aspects can be recognised. Recommended de-identification of EMR was followed in a multi-level single-use pseudonymisation scenario. Still, in practice, we decoupled health data from corresponding patient-identifying information by splitting EMR data into IDAT (identifying) and MDAT (medical). Re-identification attacks, by attribution, inference or aggregation, have not been observed in the RADAR project. Data, when transferred, always was encrypted. Present legal conditions impede realising factual anonymisation. Data minimisation was realised, together with purpose and storage limitation. For data minimisation we confined EMR data extraction to 40 predefined data fields from a small number of consenting use case-patients. These 40 variables were arranged into 11 semantic groups and correlated with the core data set of the medical informatics initiative.

**Conclusion:**

Privacy by design and data minimisation concepts are incorporated into the RADAR project and thus made it feasible in Germany. Future efforts are needed in building and maintaining a solid and transparent legal, ethical, governance and data security framework for PCP teams’ inclusion in research.

# Abstract: Baseline characteristics and external validity of older multimorbid patients with polypharmacy and general practitioners enrolled in a randomized controlled primary care trial

**DOI:** 10.1080/13814788.2021.2008352

**Authors:** Katharina Tabea Jungo, Rahel Meier, Fabio Valeri, Nathalie Schwab, Claudio Schneider, Emily Reeve, Marco Spruit, Matthias Schwenkglenks, Nicolas Rodondi, Sven Streit

**Affiliations:** Institute of Primary Health Care (BIHAM), University of Bern, Bern, Switzerland

## Abstract

**Background:**

Multimorbid older adults remain underrepresented in clinical trials, and it is challenging to recruit general practitioners and their patients for trials.

**Research question:**

This paper aims to describe the baseline characteristics of GPs and patients participating in the ‘Optimizing PharmacoTherapy in older multimorbid adults In primary CAre’ (OPTICA) trial and to compare them to reference cohorts from a Swiss real-world cohort called the ‘Family medicine ICPC Research using Electronic medical records’ (FIRE) project. It also investigates patients’ willingness to have medications deprescribed.

**Methods:**

In this cross-sectional study, 323 multimorbid (≥3 chronic conditions) patients with polypharmacy (≥5 regular medications) aged ≥65 years and 43 GPs recruited for the OPTICA trial were compared to 22,907 older multimorbid patients with polypharmacy and 227 GPs from the FIRE database. Using the revised Patients’ Attitudes Towards Deprescribing (rPATD) questionnaire, we described patients’ baseline willingness to have medications deprescribed

**Results:**

The GPs in the FIRE project and OPTICA trial were similar in terms of sociodemographic characteristics and their work as a GP (e.g. aged in their 50s, ≥10 years of experience, ≥60% self-employed, and ≥80% work in group practices). The median age of patients in the OPTICA trial was 77 years and 45% of trial participants were women. Patients participating in the OPTICA trial and patients in the FIRE database were comparable in age, specific clinical characteristics (e.g. systolic blood pressure, body mass index) and health services use (e.g. selected lab and vital data measurements). More than 80% of older multimorbid patients reported willingness to stop ≥1 of their medications if their doctor said this was possible.

**Conclusion:**

The characteristics of patients and GPs recruited to the OPTICA trial are relatively comparable to characteristics of a real-world population, indicating that the findings of the OPTICA trial will have good external validity. Most patients were open to deprescribing.

# Abstract: How does triage by an electronic symptom checker match with triage by a nurse in primary care?

**DOI:** 10.1080/13814788.2021.2008352

**Authors:** Tuomas Koskela, Ville Liu, Minna Kaila

**Affiliations:** Faculty of Medicine and Health Technology, Tampere University, Tampere, Finland

## Abstract

**Background:**

In Finland, Omaolo^©^ electronic symptom checkers (ESCs) were developed to triage primary care patients. Based on analysis of the patient’s responses to a standard set of questions, the ESC classifies him/her as emergent, urgent, not urgent, or advice on self-care. At present, there are 16 ESCs in use and integrated into primary care electronic services. The user (patient) answers questions about their symptoms on the Omaolo^©^ website and gets triage guidance. The idea is to help the user more adequately assess their condition and ease the professionals’ triage workload. To our knowledge, ESCs have previously been studied only by using standardised patient vignettes.

**Research question:**

How does triage by an ESC match with triage by a nurse? How safe is triage by ESC?

**Methods:**

Patients were recruited in primary health care waiting rooms. They were there asked to use the ESC to triage themselves and then taken to the nurse, who did the same without knowing the ESC guidance. After the triage decision, the nurse accessed the ESC guidance and commented on if that case was a mismatch. The participating nurses were experienced in triage for at least 2 years. Data on 14 different ESCs were collected in ten primary health care centres in 2019–2020.

**Results:**

For preliminary results, 700 cases were explored. In 55% of cases, the triage was exactly the same by the nurse and the symptom checker. Out of 189 cases classified as urgent by the nurse, 25 were classified as not critical or as self-care by ESC. We explored the cases in detail in which patient safety was possibly threatened but found no need to change the algorithm. The final results will be displayed in the congress.

**Conclusion:**

Patient safety was not threatened when using the ESC triage although the percentage of matches was relatively low.

# Abstract: How do patients rate the importance of parameters considered patient-relevant in recent studies? A cross-sectional survey among German general practice patients

**DOI:** 10.1080/13814788.2021.2008352

**Authors:** Christine Kersting, Julia Hülsmann, Klaus Weckbecker, Achim Mortsiefer

**Affiliations:** Professorship of Primary Care & Institute of General Practice and Interprofessional Care, Faculty of Health, Witten/Herdecke University, Witten, Germany

## Abstract

**Background:**

To make informed choices based on their individual preferences, patients need to be adequately informed about treatment options and their potential effects. This implies that studies determine the effects of care based on parameters that are relevant to patients. Our scoping review of recent studies revealed a wide variety of parameters considered relevant to patients, which addressed processes as well as outcomes of care. It is unclear which of them are most important for patients across diseases.

**Research question:**

How do patients rate the importance of process- and outcome-related parameters considered patient-relevant in previous studies?

**Methods:**

The study was designed as cross-sectional survey among German general practice patients. Ten teaching and research practices of the Institute of General Practice and Interprofessional Care supported the study. During 2 weeks in fall 2020, patients willing to participate self-administered a short questionnaire. The questionnaire evaluated 32 parameters considered patient-relevant in previous studies on a 5-point Likert scale ranging from ‘not relevant’ to ‘highly relevant’ and offered a free-text field for additional parameters. Two researchers (CK, JH) inductively categorised the free-text answers, while quantitative data was analysed using descriptive statistics in SPSS.

**Results:**

Data from 299 patients was eligible for analysis. The parameter ‘confidence in therapy’ was rated most important, followed by ‘prevention of comorbidity’ and ‘mobility.’ Overall, all parameters except ‘sexuality/sexual function’ and ‘frequency of healthcare service utilisation’ were rated important or highly important. Free-text analyses revealed 16 new parameters. Those most frequently mentioned were ‘confidence in practitioner,’ ‘inclusion of alternative medicine/treatment methods,’ ‘enough time in physician consultation,’ and ‘being heard.’

**Conclusion:**

Parameters addressing both processes and outcomes of care have a high significance for patients. Interestingly, the newly mentioned parameters predominantly addressed processes of care. Further research is needed to understand how patients set priorities and balance between parameters to make choices.

# Abstract: Premature ejaculation in primary care: communication strategies versus usual care for male patients consulting for a sexual, urogenital or psychological reason; The GET UP cluster randomised controlled trial

**DOI:** 10.1080/13814788.2021.2008352

**Authors:** Marie Barais, Marine Costa, Camille Montalvo, Vincent Rannou, Hélène Vaillant Roussel, David Costa, Sébastien Cadier, Bruno Pereira

**Affiliations:** Departement Universitaire de Médecine Générale, Université de Bretagne Occidentale, Brest Cedex, France

## Abstract

**Background:**

Premature ejaculation is the most common sexual dysfunction among men. A previous qualitative study identified six communication strategies described by general practitioners (GP) who had tackled the topic with their patients during consultations.

**Research question:**

Is training in communication skills implementing these six strategies more effective than usual care on the incidence of patients bringing up the topic of premature ejaculation with their GP?

**Methods:**

Cluster randomized controlled trial, stratified over four geographical areas comparing an intervention group that received a training session on the six strategies, and a control group that provided routine medical care. Participants were male patients between 18 and 80 years old and those consulting for a sexual, urogenital or psychological reason were included. The intervention was a communication skill training session for GPs on using the six strategies identified in the previous qualitative study. The primary outcome was the efficacy of the training session in communication skills compared with usual care, evaluated as the percentage of patients who discussed the topic of premature ejaculation with their GP. The secondary objectives were: (i) percentage of enrolled patients with premature ejaculation (identified by a score >9 of the Premature Ejaculation Diagnostic Tool filled in 4 weeks after the consultation); and (ii) variation in the quality of life (SF-12 scale score) of the enrolled patients between baseline and week 4 after the consultation for a sexual, urogenital or psychological reason.

**Results:**

130 patients were included by 32 GPs (*n* = 16 in the intervention group and *n* = 16 in the control group). The number of enrolled patients who discussed premature ejaculation was higher in the intervention group than in the control group (42% vs. 4.9%, absolute difference =37%, 95%CI [24–50%], *p* < 0.001).

**Conclusion:**

Training GPs in communication strategies to talk about premature ejaculation improves its detection.

# Abstract: Reducing unnecessary emergency department admissions in children with respiratory tract infection symptoms: A controlled educational intervention study from Turkey

**DOI:** 10.1080/13814788.2021.2008352

**Authors:** İkbal Hümay Arman, Kübra Temel Aslan, Yusuf Arman, Cigdem Apaydin Kaya

**Affiliations:** Department of Family Medicine, Marmara University School of Medicine, İstanbul, Turkey

## Abstract

**Background:**

Respiratory tract infections (RTI), the most common cause of emergency admissions in children, can mostly be managed in Family Health Centers (FHC). This requires the right attitude towards RTI symptoms (RTIS).

**Research question:**

Can education of mothers reduce unnecessary emergency admissions of children with RTIS and improve their knowledge, attitudes and behaviours (KAB) about RTIS (KABaRTIS)?

**Methods:**

This study was a controlled educational intervention in mothers of 6 months to 6-year-old children. The sample size was calculated as at least 58 + 58 and reached with convenience sampling. The intervention group was given one-on-one, face-to-face training and a brochure prepared by the researchers. RTIS management and alarm indicators were emphasised. Likert-type questions about KABaRTIS, fever-related practices, health services received by their children, number and reasons of applications were compared before (B) and after (A) intervention in control (C) and intervention (I) groups. The scale’s Cronbach-α coefficient was 0.68–0.72.

**Results:**

223 mothers participated but it was completed with 178 people (C = 118, I = 60). There was no significant difference between the groups before the intervention. KAB scores of both groups increased after the intervention (C = B: 76.9-A: 82.2; I = B: 76.9-A: 83.6; *p* < 0.001) but the difference between the two groups was not significant (*p* = 0.193). In both groups, emergency admissions for RTIS decreased, but the C-group went to the emergency for RTIS less (*p* = 0.014/*p* = 0.492). The median number of admissions decreased for both groups (B: 1-A: 0, *p* = 0.180). However, emergency department applications due to the severity of symptom/disease increased in C-group and decreased in I-group.

**Conclusion:**

There may be a few reasons for not seeing the expected difference between the intervention and control group after the intervention. The study population went to emergency departments less than the average Turkish population. The training was done in one session. Educational content may have increased the sensitivity of mothers in the I-group. This is an important study because it is the first educational intervention planned in Turkey to reduce unnecessary emergency admissions in children. However, further long-term studies are needed.

# Abstract: Immediate or delayed prescription of antibiotics – Factors that influence general practitioner decision

**DOI:** 10.1080/13814788.2021.2008352

**Authors:** Katarina Stavrikj, Valentina Risteska - Nejasmic, Aleksandar Kirkovski, Nikica Jovanovski, Martin Angelov, Bekim Ismaili, Marta Tundzeva

**Affiliations:** Medical Faculty, Center for Family Medicine, Skopje, North Macedonia

## Abstract

**Background:** Respiratory tract infections are one of the most common causes of antibiotic prescription in primary care. The inappropriate and overuse of antibiotics leads to the increased emergence of antimicrobial resistance.

**Research question:** What are the factors that influence the prescription pattern of antimicrobials in patients with acute respiratory tract infections in primary care in Macedonia.

**Methods:** A cross-sectional study, based on a representative sample of 87 general practitioners (GPs), was conducted during 4 weeks in November 2019. All patients with an episode of acute respiratory infection (ARI) were involved in the survey. We used a special questionnaire that was used during the same survey in 2014/2015. The association between diagnosis, patient sociodemographic factors and manner of antibiotic prescription (immediate or delayed prescription) was investigated. The programme Statistica was used for statistical analysis.

**Results:** Antibiotics were prescribed to 4237 patients (53%) with ARI. GPs used immediate prescription for 2878 patients (77.39%) while delaying prescription for 841 patients (22.61%).

The immediate prescription was more often used for the diagnoses J03 Tonsillitis 32.45%, J20 Bronchitis 19.74% and J02 Pharyngitis 16.33%. The delayed prescription was more often used for J03 Tonsillitis 24.39%, J02 Pharyngitis 21.64% and J20 Bronchitis 20.33%. Multiple regression analysis was run to predict the prescription manner of antibiotics. Lower age, workday–Friday and follow-up visit statistically significantly predicted delayed prescription of antibiotics (*p* < 0.05). Factors influencing doctors’ perceptions around the delayed prescription of antibiotics were mainly difficulties with follow up (20.33%), presence of complication (14.39%), patient pressure (11.41%) and uncertainty in diagnosis (6.66%) while for immediate prescription it was an indication for antibiotics (79.95%).

**Conclusion:** Exploring the factors that influence the doctor’s decision for immediate or delayed prescription of antibiotics for ARI is important for improving antibiotic stewardship in primary care and closing the gap in the evidence base.

# Abstract: Association of sense of coherence and depression in patients with chronic pain: A systematic review and meta-analysis

**DOI:** 10.1080/13814788.2021.2008352

**Authors:** Alejandra Aguilar-Latorre, Bárbara Oliván Blázquez, Ángela Asensio-Martínez, Celia Álvarez Bueno, Iván Cavero Redondo, Magallon Botaya

**Affiliations:** Institute for Health Research Aragón (IIS Aragón), Zaragoza, Spain

## Abstract

**Background:**

Chronic pain is a significant complaint that generates distressing consequences for those who suffer it. Pain and depression concur in a context of comorbidity and both share underlying stress conditions. Sense of coherence (SOC) is a factor that determines how well a person manages stress and stays healthy. Its relationship with depression is highly reported.

**Research question:**

Our objective was to assess the size of the available evidence on the association of SOC with depression in patients with chronic pain.

**Methods:**

A systematic review and meta-analysis were performed. Searches were conducted from 01 April to 31 May 2020, in PubMed, Web of Science, Embase, PsycINFO, Psicodoc, ScienceDirect and Dialnet. The evidence regarding the relationship between SOC and depression in patients with chronic pain was summarised and compared.

**Results:**

162 articles were identified. Ten studies were included in the qualitative analysis and nine in the quantitative analysis. The pooled correlation coefficient was -0.55 (95%: −0.70; −0.41). The heterogeneity across the studies was considerable (*I*^2^ = 94.8%; *p* < 0.001). The sensitivity analysis showed that the pooled correlation coefficient was not modified after removing any study. The random‐effects meta‐regression models for the association between SOC and depression showed that age (*p* = 0.148) and the percentage of women (*p* = 0.307) were not related to heterogeneity across studies. No publication bias was detected (*p* = 0.720).

**Conclusion:**

The included studies indicate that SOC is an important factor in depression levels in chronic pain patients. Most of the included studies showed a moderate association between SOC and depressive symptoms. Our results, therefore, present implications for the design of public health policies and rehabilitation programmes. Adopting a systematic salutogenic orientation to focus on cognitive and emotional resources that are available, strengthening them and creating new ones would be very useful.

# Abstract: Towards a core curriculum for Italian family medicine: Results of a transparent, participatory and collective writing.

**DOI:** 10.1080/13814788.2021.2008352

**Authors:** Alice Serafini, Stefano Celotto, Jacopo Demurtas, Giuseppe Febbo, Davide Luppi, Alessandro Mereu, Nicola Pecora, Giorgio Sessa, Luigi Brachitta, Peter Konstantin Kurotschka, Ilaria Rossiello, Elena Rubatto

**Affiliations:** Local Health Service of Modena, Modena, Italy

## Abstract

**Background:**

Scientific evidence shows how Family Medicine (FM), internationally recognised as an academic and scientific discipline, has a significant impact on the health of the community and the appropriate use of public resources. Despite this, in Italy, the national legislation of reference for FM training does not define the educational programmes to be followed, competencies to be acquired, teaching and learning methods of the discipline or the criteria of evaluation for the achievement of the title of a general practitioner. A project for writing a Core Curriculum of the Italian GP and the dedicated working group ‘MMG Core Curriculum Task Force’ have been promoted by the Movimento Giotto (MG, member of the Vasco da Gama Movement).

**Research question:**

To elaborate a Core Curriculum that establishes what, where, how to learn and how to evaluate the specialised competencies of professional FM.

**Methods:**

From February to May 2019, a transparent process of collective and participatory writing took place, through different phases: An internal call through the MG for the recruitment of the Coordinator’s Task Force (9 coordinators); self-training and drafting of the index of the document, from a comparative perspective; a public call to recruit collaborators (52 young Italian FMs) for the collective drawing up of the chapters; a peer review of the contents produced.

**Results:**

A 200-page document, ‘Towards the Core Curriculum of General Medicine’ has been produced, divided into three chapters describing the rationale of the project, the Core Competencies, and the clinical skills identified in five macro-areas of clinical practice.

**Conclusion:**

The proposal document, thus necessarily still incomplete, needs a deeper reflection and sharing with other health system actors. The MG aims to proceed to further steps in the coming months to build consent on the document (Delphi Method or Consensus Conference).

# Abstract: Translation and validation of two Parkinson’s disease-specific burden questionnaires, for patients and caregivers, from German into French

**DOI:** 10.1080/13814788.2021.2008352

**Authors:** Sabine Bayen, Jean Heutte, Jean-Charles Vanderbecken, Caroline Moreau, Luc Defebvre, Wassil Messaadi, David Devos, Nassir Messaadi

**Affiliations:** Department of General Practice, University of Lille, Lille Cedex, France

## Abstract

**Background:** Parkinson’s Disease (PD) is a common neurodegenerative condition. Its various clinical expression affects patients’ and caregivers’ psycho-social wellbeing. Two specific questionnaires exist in German to assess the PD-related psycho-social burden in a dyad (patient and spouse). The Bela-A-K and Bela-P-K, questionnaires allow healthcare professionals to cross-check the patient’s (Bela-P-K) and the spouse’s (Bela-A-K) perspective of wellbeing despite PD. Four dimensions are explored: physical performance, emotional charge, social relationships, and couple/family life. The study’s purpose was to translate, test among French patients and spouses, and validate the translated versions.

**Research question:** How strong are the questionnaires’ internal consistency and temporal stability?

**Methods:** The questionnaires were translated from German into French, using forwards-and-backwards translation, followed by a cultural cross-check. The participants were recruited by a GP and PD-nurses and invited to test the French version in its online administered version, created via the Lime Survey^®^ software. The participants had to fill out the questionnaires twice to respect the ‘test–retest’ method. 5 days after their first assessment, they answered again. Data analysis was performed using SPSS software.

**Results:** Nineteen couples participated in the study: 18 patients (14 M/4F); 18 spouses (4M/14F).

The Bela-A-K showed strong temporal stability, whereas it was weak for the dimension of social relationships. The Bela-P-K showed a strong internal consistency but significant differences for ten items between the moment of test and retest. Some items of the Bela-P-K must be simplified to improve its temporal stability, considering the patient’s changing form on the day. The items related to the dimension of social relationships must be adjusted for the Bela-A-K.

**Conclusion:** The questionnaires are useful and reliable. They contribute to a positive psychological dyad-centred follow-up and positive health education in the case of PD.

# Abstract: Rock-a-bye baby: Children’s sleep habits and related factors

**DOI:** 10.1080/13814788.2021.2008352

**Authors:** Ece Saykan Balatacı, Gizem Limnili, Nilgün Özçakar

**Affiliations:** Faculty of Medicine, Dokuz Eylul University, Izmir, Turkey

## Abstract

**Background:**

Adequate sleep is vital for both healthy development and optimum daytime functioning in children of all ages. School periods in which positive preschool behaviors are reinforced are essential for child development. Inadequate sleep in school-aged children harms mood, attention and school performance.

**Research question:**

What are the sleeping habits and related factors of school-aged children in Izmir, Turkey?

**Methods:**

1152 parents who have an elementary school-aged child were enrolled in this cross-sectional study. A form that consists of children’s sociodemographic features, Children’s Sleep Habits Questionnaire (CSHQ) and Strength and Difficulties Questionnaire (SDQ) was used.

**Results:**

Of the participants, 68.9% have sleeping problems according to the CSHQ. Sleep problems were frequent if the child does not have a sibling, is in an upper grade at school, sleeps late, watches TV before sleeping, does not read a book before sleeping, has behavioural and emotional problems or attention-deficit/hyperactivity disorder. Attention-deficit/hyperactivity disorder, emotional problems, peer problems and behavioural problems were significantly higher in those with sleep problems (*p* < 0.001).

**Conclusion:**

Sleeping problems would decrease if parents are informed about sleep hygiene, regular and appropriate sleep, and if families and physicians are educated about emotional and behavioural problems that may accompany sleeping problems.
